# The genome sequence of a fungus weevil, *Platystomos albinus *(C.Linnaeus, 1758)

**DOI:** 10.12688/wellcomeopenres.24290.1

**Published:** 2025-06-02

**Authors:** Liam M. Crowley, Xavier Richard Badham, University of Oxford and Wytham Woods Genome Acquisition Lab, Darwin Tree of Life Barcoding collective, Wellcome Sanger Institute Tree of Life Management, Samples and Laboratory team, Wellcome Sanger Institute Scientific Operations: Sequencing Operations, Wellcome Sanger Institute Tree of Life Core Informatics team, Tree of Life Core Informatics collective, Darwin Tree of Life Consortium

**Affiliations:** 1 Department of Biology, University of Oxford, Oxford, England, UK; 2 Queen's University Belfast, Belfast, Northern Ireland, UK; 3 University of Aberdeen, Aberdeen, Scotland, UK

**Keywords:** Platystomos albinus, fungus weevil, genome sequence, chromosomal, Coleoptera

## Abstract

We present a genome assembly from a female specimen of *Platystomos albinus* (fungus weevil; Arthropoda; Insecta; Coleoptera; Anthribidae). The assembly contains two haplotypes with total lengths of 555.31 megabases and 554.55 megabases. Haplotype 1 is scaffolded into 11 chromosomal pseudomolecules, including the X sex chromosome. Haplotype 2 was assembled to scaffold level. The mitochondrial genome has also been assembled, with a length of 17.09 kilobases.

## Species taxonomy

Eukaryota; Opisthokonta; Metazoa; Eumetazoa; Bilateria; Protostomia; Ecdysozoa; Panarthropoda; Arthropoda; Mandibulata; Pancrustacea; Hexapoda; Insecta; Dicondylia; Pterygota; Neoptera; Endopterygota; Coleoptera; Polyphaga; Cucujiformia; Curculionoidea; Anthribidae; Anthribinae; Platystomini; *Platystomos*; *Platystomos albinus* (C.Linnaeus, 1758) (NCBI:txid197009)

## Background

*Platystomus albinus* (Linnaeus, 1758) is a species of beetle within the family Anthribidae, which is commonly referred to as the fungus weevils. Members of this family, including *P. albinus*, are typically associated with decaying wood and fungal growth, where they play roles in nutrient cycling and decomposition ( Arnett *et al.*, 2002).

This species has a broad distribution ranging from Western Europe to Western Siberia, excluding the Mediterranean and similar relatively arid regions, where it is largely absent ( GBIF Secretariat, 2023). Within Great Britain and Ireland, its range is restricted to southern England and Wales, though recent, unverified reports suggest that it may also be present in parts of western Ireland ( NBN Atlas Partnership, 2024). In Great Britain, adult beetles are most frequently observed between August and October.

The larvae of *P. albinus* are saproxylic, feeding on decaying wood and typically associated with standing dead trees ( Alexander, 2002). Common host trees for larvae include species such as beech ( *Fagus sylvatica*), hazel ( *Corylus avellana*), and alder ( *Alnus glutinosa*). Adults of the species tend to remain at the site where larvae emerge ( Gønget, 2003), but naturally are less limited in range. Anthribidae have been previously reported to have associations with specific fungal species and *P. albinus* has been claimed to have an association with *Daldinia* fungi, but these associations have never been verified ( Arnett *et al.*, 2002).

*Platystomos albinus* adults are among the larger British weevils with a body length ranging from 7 mm to 12 mm long. The body of *P. albinus* is mottled black or brown aside from bright white markings. These are distributed on the dorsal surface of the rostrum, the apex of the elytra both dorsally and ventrally, and in white patches on the mid-section of each elytron. There are alternating white and black stripes along the antennae and legs. It has been suggested that this colouring pattern mimics bird droppings against wood. The species if sexually dimorphic with the female typically larger and broader with antennae roughly as long as the head and thorax, whereas the male antennae are approximately the length of its body. Superficially, *Platystomos albinus* is similar to *Platyrhinus resinosus* and the two are often found in close proximity. However, *P. resinosus* exhibits a broader rostrum, duller white patches and more complex elytral patterning.

**Figure 1.**
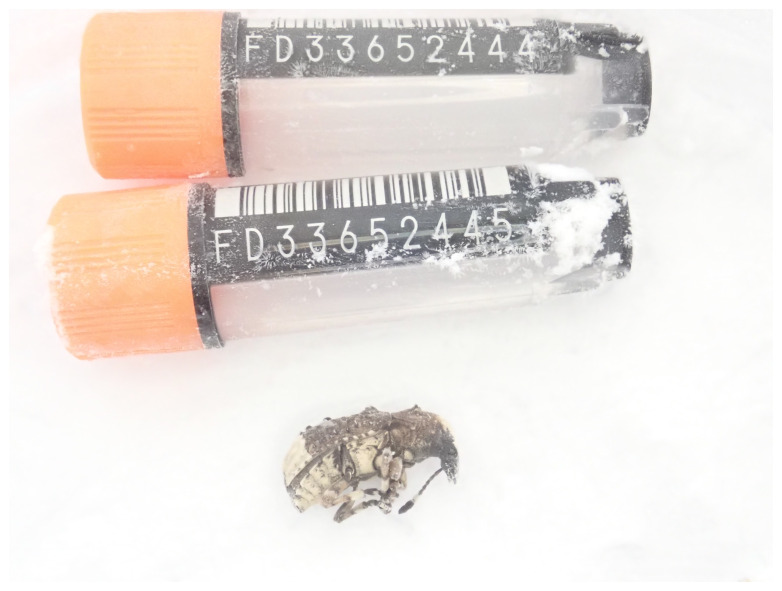
Photograph of the *Platystomos albinus* (icPlaAlbi1) specimen used for genome sequencing.

The genome of *Platystomus albinus* ( Figure 1) was sequenced as part of the Darwin Tree of Life Project, a collaborative effort to sequence all named eukaryotic species in the Atlantic Archipelago of Britain and Ireland.

## Genome sequence report

### Sequencing data

The genome of a specimen of *Platystomos albinus* ( Figure 1) was sequenced using Pacific Biosciences single-molecule HiFi long reads, generating 24.96 Gb (gigabases) from 2.40 million reads, which were used to assemble the genome. GenomeScope analysis estimated the haploid genome size at 580.98 Mb, with a heterozygosity of 1.75% and repeat content of 47.27%. These estimates guided expectations for the assembly. Based on the estimated genome size, the sequencing data provided approximately 41 coverage. Hi-C sequencing produced 117.73 Gb from 779.65 million reads, used to scaffold the assembly. RNA sequencing data were also generated and are available in public sequence repositories. Table 1 summarises the specimen and sequencing details.

**Table 1.** Specimen and sequencing data for *Platystomos albinus*.

Project information			
**Study title**	Platystomos albinus		
**Umbrella BioProject**	PRJEB75287		
**Species**	*Platystomos albinus*		
**BioSpecimen**	SAMEA112232584		
**NCBI taxonomy ID**	197009		
Specimen information			
**Technology**	**ToLID**	**BioSample accession**	**Organism part**
**PacBio long read sequencing**	icPlaAlbi1	SAMEA112233044	head and thorax
**Hi-C sequencing**	icPlaAlbi1	SAMEA112233045	abdomen
**RNA sequencing**	icPlaAlbi1	SAMEA112233045	abdomen
Sequencing information			
**Platform**	**Run accession**	**Read count**	**Base count (Gb)**
**Hi-C Illumina NovaSeq X**	ERR12982570	7.80e+08	117.73
**PacBio Sequel IIe**	ERR12954127	2.40e+06	24.96
**RNA Illumina NovaSeq X**	ERR14792831	9.98e+07	15.07

### Assembly statistics

The genome was assembled into two haplotypes using Hi-C phasing. Haplotype 1 was curated to chromosome level, while haplotype 2 was assembled to scaffold level. The assembly was improved by manual curation, which corrected 110 misjoins or missing joins and removed 61 haplotypic duplications. These interventions decreased the scaffold count by 90.24%. The final assembly has a total length of 555.31 Mb in 11 scaffolds, with 250 gaps, and a scaffold N50 of 69.36 Mb ( Table 2).

**Table 2.** Genome assembly data for *Platystomos albinus*.

Genome assembly	Haplotype 1	Haplotype 2
Assembly name	icPlaAlbi1.hap1.1	icPlaAlbi1.hap2.1
Assembly accession	GCA_964106875.1	GCA_964106955.1
Assembly level	chromosome	scaffold
Span (Mb)	555.31	554.55
Number of contigs	261	302
Number of scaffolds	11	123
Longest scaffold (Mb)	129.7	-
Assembly metrics (benchmark)	Haplotype 1	Haplotype 2
Contig N50 length (≥ 1 Mb)	4.33 Mb	4.81 Mb
Scaffold N50 length (= chromosome N50)	69.36 Mb	68.52 Mb
Consensus quality (QV) (≥ 40)	62.3	62.0
*k*-mer completeness	69.85%	69.74%
Combined *k*-mer completeness (≥ 95%)	98.47%	
BUSCO * (S > 90%; D < 5%)	C:98.8%[S:98.3%,D:0.5%], F:0.3%,M:0.9%,n:2,124	-
Percentage of assembly assigned to chromosomes (≥ 90%)	100.0%	-
Sex chromosomes (localised homologous pairs)	X	-
Organelles (one complete allele)	Mitochondrial genome: 17.09 kb	-

*BUSCO scores based on the endopterygota_odb10 BUSCO set using version 5.5.0. C = complete [S = single copy, D = duplicated], F = fragmented, M = missing, n = number of orthologues in comparison.

**Figure 2.**
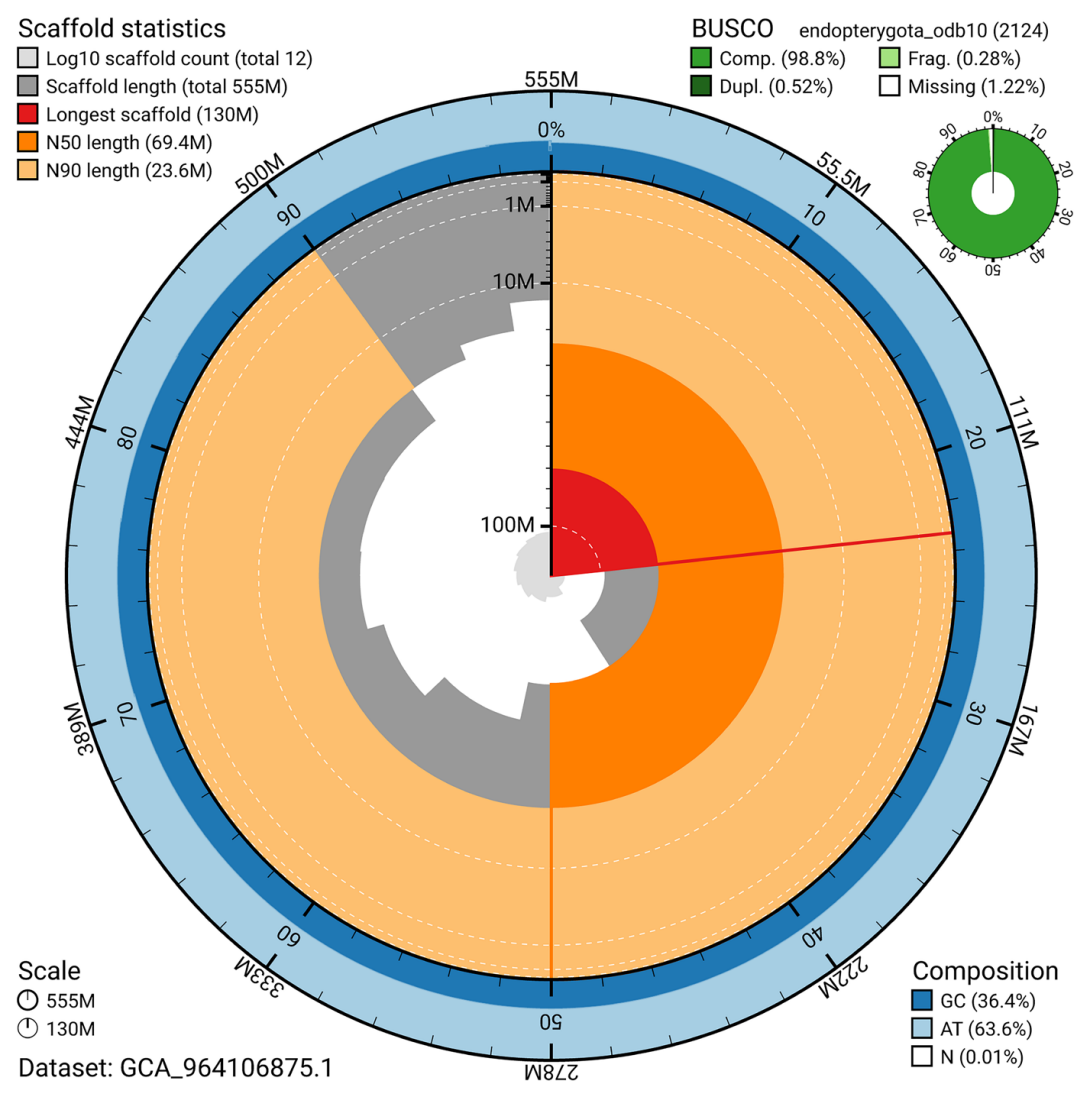
Genome assembly of *Platystomos albinus*, icPlaAlbi1.hap1.1: metrics. The BlobToolKit snail plot provides an overview of assembly metrics and BUSCO gene completeness. The circumference represents the length of the whole genome sequence, and the main plot is divided into 1,000 bins around the circumference. The outermost blue tracks display the distribution of GC, AT, and N percentages across the bins. Scaffolds are arranged clockwise from longest to shortest and are depicted in dark grey. The longest scaffold is indicated by the red arc, and the deeper orange and pale orange arcs represent the N50 and N90 lengths. A light grey spiral at the centre shows the cumulative scaffold count on a logarithmic scale. A summary of complete, fragmented, duplicated, and missing BUSCO genes in the endopterygota_odb10 set is presented at the top right. An interactive version of this figure is available at https://blobtoolkit.genomehubs.org/view/GCA_964106875.1/dataset/GCA_964106875.1/snail.

**Figure 3.**
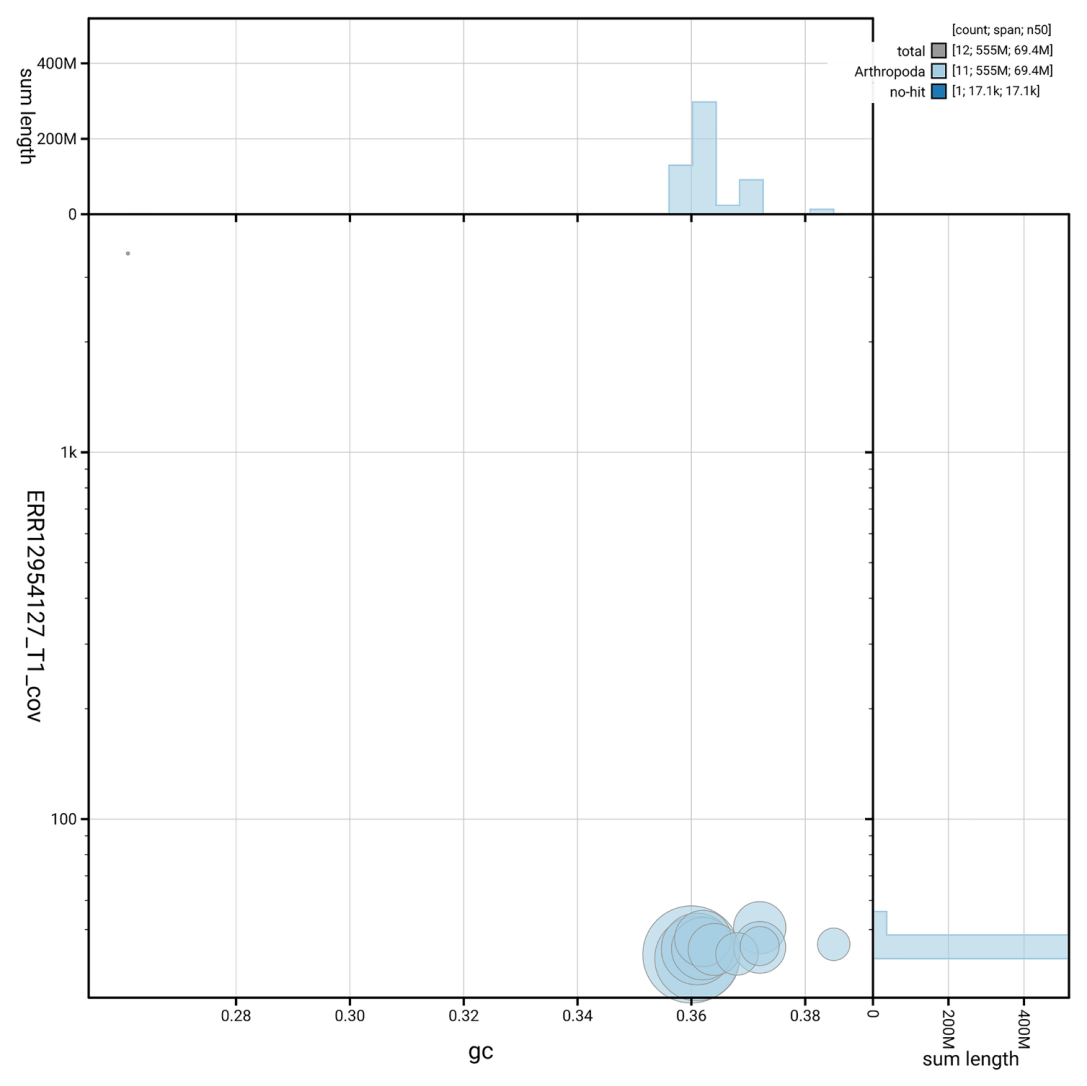
Genome assembly of *Platystomos albinus*, icPlaAlbi1.hap1.1: BlobToolKit GC-coverage plot. Blob plot showing sequence coverage (vertical axis) and GC content (horizontal axis). The circles represent scaffolds, with the size proportional to scaffold length and the colour representing phylum membership. The histograms along the axes display the total length of sequences distributed across different levels of coverage and GC content. An interactive version of this figure is available at https://blobtoolkit.genomehubs.org/view/GCA_964106875.1/dataset/GCA_964106875.1/blob.

**Figure 4.**
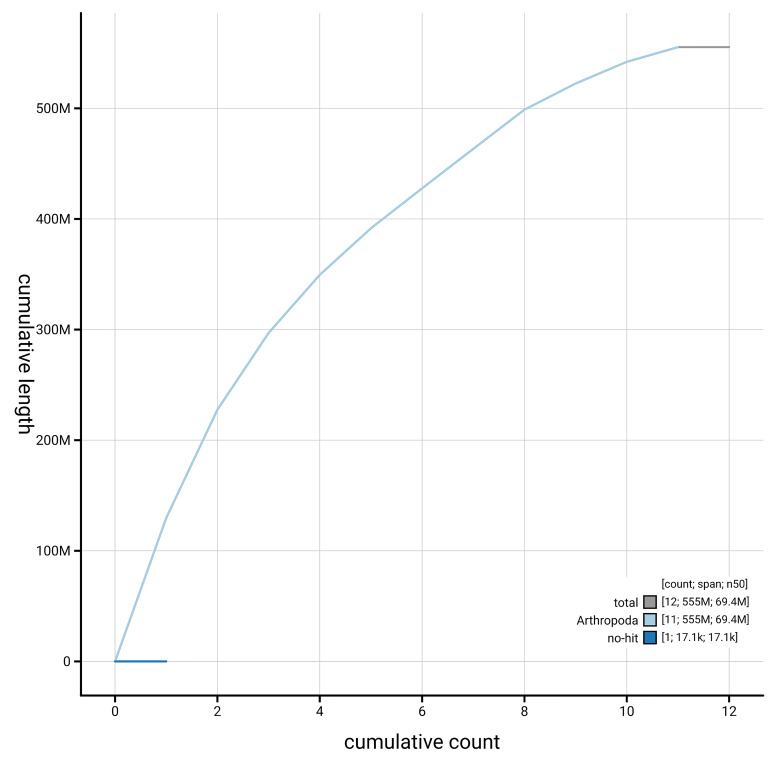
Genome assembly of *Platystomos albinus,* icPlaAlbi1.hap1.1: BlobToolKit cumulative sequence plot. The grey line shows cumulative length for all scaffolds. Coloured lines show cumulative lengths of scaffolds assigned to each phylum using the buscogenes taxrule. An interactive version of this figure is available at https://blobtoolkit.genomehubs.org/view/GCA_964106875.1/dataset/GCA_964106875.1/cumulative.

The snail plot in Figure 2 provides a summary of the assembly statistics, indicating the distribution of scaffold lengths and other assembly metrics. Figure 3 shows the distribution of scaffolds by GC proportion and coverage. Figure 4 presents a cumulative assembly plot, with separate curves representing different scaffold subsets assigned to various phyla, illustrating the completeness of the assembly.

**Figure 5.**
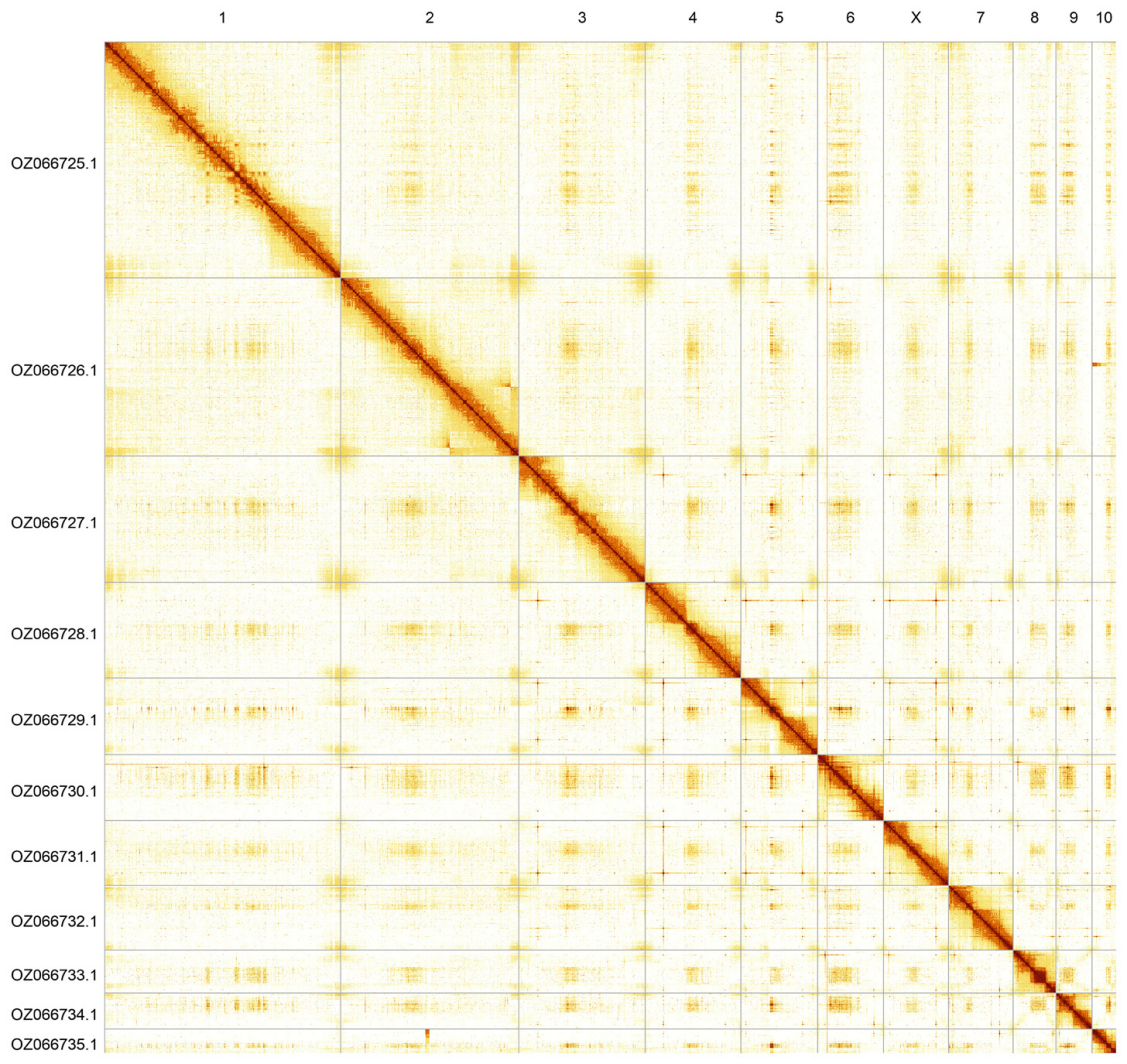
Genome assembly of *Platystomos albinus*. Hi-C contact map of the icPlaAlbi1.hap1.1 assembly, generated using PretextSnapshot. Chromosomes are shown in order of size and labelled with chromosome numbers (top) and chromosome accession numbers (left).

The whole assembly sequence was assigned to 11 chromosomal-level scaffolds, representing 10 autosomes and the X sex chromosome. These chromosome-level scaffolds, confirmed by Hi-C data, are named according to size ( Figure 5; Table 3). During curation, the sex chromosome (X) was assigned based on homology to the genome of *Pseudeuparius sepicola* (GCA_963920635.1) ( Booth *et al.*, 2024). A haplotypic inversion was observed on chromosome 2 in the region of 60.17–92.54 Mbp.

**Table 3.** Chromosomal pseudomolecules in the genome assembly of *Platystomos albinus*, icPlaAlbi1.

INSDC accession	Name	Length (Mb)	GC%
OZ066725.1	1	129.7	36
OZ066726.1	2	97.86	36
OZ066727.1	3	69.36	36
OZ066728.1	4	52.54	36
OZ066729.1	5	42.1	36
OZ066730.1	6	36.13	37
OZ066732.1	7	35.46	37
OZ066733.1	8	23.58	37
OZ066734.1	9	19.65	37
OZ066735.1	10	13.26	38.5
OZ066731.1	X	35.67	36.5
OZ066736.1	MT	0.02	26.5

The mitochondrial genome was also assembled. This sequence is included as a contig in the multifasta file of the genome submission and as a standalone record.

### Assembly quality metrics

The estimated Quality Value (QV) and *k*-mer completeness metrics, along with BUSCO completeness scores, were calculated for each haplotype and the combined assembly. The QV reflects the base-level accuracy of the assembly, while *k*-mer completeness indicates the proportion of expected *k*-mers identified in the assembly. BUSCO scores provide a measure of completeness based on benchmarking universal single-copy orthologues.

For haplotype 1, the estimated QV is 62.3, and for haplotype 2, 62.0. When the two haplotypes are combined, the assembly achieves an estimated QV of 62.1. The *k*-mer completeness is 69.85% for haplotype 1 and 69.74% for haplotype 2; and 98.47% for the combined haplotypes. BUSCO v.5.5.0 analysis using the endopterygota_odb10 reference set ( *n* = 2,124) identified 98.8% of the expected gene set (single = 98.3%, duplicated = 0.5%) for haplotype 1.

Table 2 provides assembly metric benchmarks adapted from Rhie *et al.* (2021) and the Earth BioGenome Project Report on Assembly Standards September 2024. The haplotype 1 assembly achieves the EBP reference standard of **6**. **C.Q62**.

## Methods

### Sample acquisition and DNA barcoding

The specimen used for genome sequencing was an adult female *Platystomos albinus* (specimen ID Ox002365, ToLID icPlaAlbi1), collected from Wytham Woods, Oxfordshire, United Kingdom (latitude 51.77, longitude –1.336) on 2022-05-21 by potting. The specimen was collected and identified by Liam Crowley (University of Oxford) and preserved on dry ice.

The initial identification was verified by an additional DNA barcoding process according to the framework developed by Twyford *et al.* (2024). A small sample was dissected from the specimen and stored in ethanol, while the remaining parts were shipped on dry ice to the Wellcome Sanger Institute (WSI) ( Pereira *et al.*, 2022). The tissue was lysed, the COI marker region was amplified by PCR, and amplicons were sequenced and compared to the BOLD database, confirming the species identification ( Crowley *et al.*, 2023). Following whole genome sequence generation, the relevant DNA barcode region was also used alongside the initial barcoding data for sample tracking at the WSI ( Twyford *et al.*, 2024). The standard operating procedures for Darwin Tree of Life barcoding have been deposited on protocols.io ( Beasley *et al.*, 2023).

Metadata collection for samples adhered to the Darwin Tree of Life project standards described by Lawniczak *et al*. (2022).

### Nucleic acid extraction

The workflow for high molecular weight (HMW) DNA extraction at the Wellcome Sanger Institute (WSI) Tree of Life Core Laboratory includes a sequence of procedures: sample preparation and homogenisation, DNA extraction, fragmentation and purification ( Howard *et al.*, 2025). Detailed protocols are available on protocols.io ( Denton *et al.*, 2023b). The icPlaAlbi1 sample was prepared for DNA extraction by weighing and dissecting it on dry ice ( Jay *et al.*, 2023). Tissue from the head and thorax was homogenised using a PowerMasher II tissue disruptor ( Denton *et al.*, 2023a).

HMW DNA was extracted in the WSI Scientific Operations core using the Automated MagAttract v2 protocol ( Oatley *et al.*, 2023). The DNA was sheared into an average fragment size of 12–20 kb in a Megaruptor 3 system ( Bates *et al.*, 2023). Sheared DNA was purified by solid-phase reversible immobilisation, using AMPure PB beads to eliminate shorter fragments and concentrate the DNA ( Strickland *et al.*, 2023). The concentration of the sheared and purified DNA was assessed using a Nanodrop spectrophotometer and Qubit Fluorometer using the Qubit dsDNA High Sensitivity Assay kit. Fragment size distribution was evaluated by running the sample on the FemtoPulse system.

RNA was extracted from abdomen tissue of icPlaAlbi1 in the Tree of Life Laboratory at the WSI using the RNA Extraction: Automated MagMax™ *mir*Vana protocol ( do Amaral *et al.*, 2023). The RNA concentration was assessed using a Nanodrop spectrophotometer and a Qubit Fluorometer using the Qubit RNA Broad-Range Assay kit. Analysis of the integrity of the RNA was done using the Agilent RNA 6000 Pico Kit and Eukaryotic Total RNA assay.

### Hi-C sample preparation and crosslinking

Hi-C data were generated from 20–50 mg of frozen tissue from the abdomen of the icPlaAlbi1 sample using the Arima-HiC v2 kit (Arima Genomics). As per manufacturer’s instructions, tissue was fixed, and the DNA crosslinked using a TC buffer with a final formaldehyde concentration of 2%. The tissue was then homogenised using the Diagnocine Power Masher-II. The crosslinked DNA was digested using a restriction enzyme master mix, then biotinylated and ligated. A clean up was performed with SPRIselect beads prior to library preparation. DNA concentration was quantified using the Qubit Fluorometer v4.0 (Thermo Fisher Scientific) and Qubit HS Assay Kit, and sample biotinylation percentage was estimated using the Arima-HiC v2 QC beads.

### Library preparation and sequencing

Library preparation and sequencing were performed at the WSI Scientific Operations core.

***PacBio HiFi***

Samples with an average fragment size greater than 8 kb and total mass exceeding 400 ng were eligible for the low-input SMRTbell Prep Kit 3.0 protocol (Pacific Biosciences, California, USA), depending on genome size and required sequencing depth. Libraries were prepared using the SMRTbell Prep Kit 3.0 according to the manufacturer’s instructions. The kit includes reagents for end repair/A-tailing, adapter ligation, post-ligation SMRTbell bead clean-up, and nuclease treatment. Size selection and clean-up were performed using diluted AMPure PB beads (Pacific Biosciences). DNA concentration was quantified using a Qubit Fluorometer v4.0 (ThermoFisher Scientific) and the Qubit 1X dsDNA HS assay kit. Final library fragment size was assessed with the Agilent Femto Pulse Automated Pulsed Field CE Instrument (Agilent Technologies) using the gDNA 55 kb BAC analysis kit.

The sample was sequenced using the Sequel IIe system (Pacific Biosciences, California, USA). The concentration of the library loaded onto the Sequel IIe was in the range 40–135 pM. The SMRT link software, a PacBio web-based end-to-end workflow manager, was used to set-up and monitor the run, and carry out primary and secondary data analysis.

***Hi-C***

For Hi-C library preparation, the biotinylated DNA constructs were fragmented using a Covaris E220 sonicator and size-selected to 400–600 bp using SPRISelect beads. DNA was then enriched using Arima-HiC v2 Enrichment beads. The NEBNext Ultra II DNA Library Prep Kit (New England Biolabs) was used for end repair, A-tailing, and adapter ligation, following a modified protocol in which library preparation is carried out while the DNA remains bound to the enrichment beads. PCR amplification was performed using KAPA HiFi HotStart mix and custom dual-indexed adapters (Integrated DNA Technologies) in a 96-well plate format. Depending on sample concentration and biotinylation percentage determined at the crosslinking stage, samples were amplified for 10–16 PCR cycles. Post-PCR clean-up was carried out using SPRISelect beads. The libraries were quantified using the Accuclear Ultra High Sensitivity dsDNA Standards Assay kit (Biotium) and normalised to 10 ng/μL before sequencing. Hi-C sequencing was performed on the Illumina NovaSeq X instrument using 150 bp paired-end reads.

***RNA***

Poly(A) RNA-Seq libraries were prepared using the NEBNext ^®^ Ultra™ II Directional RNA Library Prep Kit for Illumina (New England Biolabs), following the manufacturer’s instructions. Poly(A) mRNA in the total RNA solution was isolated using oligo(dT) beads, converted to cDNA, and uniquely indexed; 14 PCR cycles were performed. Libraries were size-selected to produce fragments between 100–300 bp. Libraries were quantified, normalised, pooled to a final concentration of 2.8 nM, and diluted to 150 pM for loading. Sequencing was carried out on the Illumina NovaSeq X instrument.

### Genome assembly, curation and evaluation

***Assembly***

Prior to assembly of the PacBio HiFi reads, a database of *k*-mer counts ( *k* = 31) was generated from the filtered reads using FastK. GenomeScope2 ( Ranallo-Benavidez *et al.*, 2020) was used to analyse the *k*-mer frequency distributions, providing estimates of genome size, heterozygosity, and repeat content.

The HiFi reads were assembled using Hifiasm in Hi-C phasing mode ( Cheng *et al.*, 2021; Cheng *et al.*, 2022), resulting in a pair of haplotype-resolved assemblies. The Hi-C reads ( Rao *et al.*, 2014) were mapped to the primary contigs using bwa-mem2 ( Vasimuddin *et al.*, 2019). The contigs were further scaffolded with Hi-C data in YaHS ( Zhou *et al.*, 2023), using the --break option for handling potential misassemblies. The scaffolded assemblies were evaluated using Gfastats ( Formenti *et al.*, 2022), BUSCO ( Manni *et al.*, 2021) and MERQURY.FK ( Rhie *et al.*, 2020).

The mitochondrial genome was assembled using MitoHiFi ( Uliano-Silva *et al.*, 2023), which runs MitoFinder ( Allio *et al.*, 2020) and uses these annotations to select the final mitochondrial contig and to ensure the general quality of the sequence.

***Assembly curation***

The assembly was decontaminated using the Assembly Screen for Cobionts and Contaminants (ASCC) pipeline. Flat files and maps used in curation were generated via the TreeVal pipeline ( Pointon *et al.*, 2023). Manual curation was conducted primarily in PretextView ( Harry, 2022) and HiGlass ( Kerpedjiev *et al.*, 2018), with additional insights provided by JBrowse2 ( Diesh *et al.*, 2023). Scaffolds were visually inspected and corrected as described by Howe *et al*. (2021). Any identified contamination, missed joins, and mis-joins were amended, and duplicate sequences were tagged and removed. Sex chromosomes were identified by synteny analysis. The curation process is documented at https://gitlab.com/wtsi-grit/rapid-curation.

***Assembly quality assessment***

The Merqury.FK tool ( Rhie *et al.*, 2020), run in a Singularity container ( Kurtzer *et al.*, 2017), was used to evaluate *k*-mer completeness and assembly quality for both haplotypes using the *k*-mer databases ( *k* = 31) computed prior to genome assembly. The analysis outputs included assembly QV scores and completeness statistics.

The genome was analysed using the BlobToolKit pipeline, a Nextflow ( Di Tommaso *et al.*, 2017) implementation of the earlier Snakemake BlobToolKit pipeline ( Challis *et al.*, 2020). The pipeline aligns PacBio reads using minimap2 ( Li, 2018) and SAMtools ( Danecek *et al.*, 2021) to generate coverage tracks. Simultaneously, it queries the GoaT database ( Challis *et al.*, 2023) to identify relevant BUSCO lineages and runs BUSCO ( Manni *et al.*, 2021). For the three domain-level BUSCO lineages, BUSCO genes are aligned to the UniProt Reference Proteomes database ( Bateman *et al.*, 2023) using DIAMOND blastp ( Buchfink *et al.*, 2021). The genome is divided into chunks based on the density of BUSCO genes from the closest taxonomic lineage, and each chunk is aligned to the UniProt Reference Proteomes database with DIAMOND blastx. Sequences without hits are chunked using seqtk and aligned to the NT database with blastn ( Altschul *et al.*, 1990). The BlobToolKit suite consolidates all outputs into a blobdir for visualisation.

The BlobToolKit pipeline was developed using nf-core tooling ( Ewels *et al.*, 2020) and MultiQC ( Ewels *et al.*, 2016), with package management via Conda and Bioconda ( Grüning *et al.*, 2018), and containerisation through Docker ( Merkel, 2014) and Singularity ( Kurtzer *et al.*, 2017).

Table 4 contains a list of relevant software tool versions and sources.

**Table 4.** Software tools: versions and sources.

Software tool	Version	Source
BLAST	2.14.0	ftp://ftp.ncbi.nlm.nih.gov/blast/executables/blast+/
BlobToolKit	4.3.9	https://github.com/blobtoolkit/blobtoolkit
BUSCO	5.5.0	https://gitlab.com/ezlab/busco
bwa-mem2	2.2.1	https://github.com/bwa-mem2/bwa-mem2
DIAMOND	2.1.8	https://github.com/bbuchfink/diamond
fasta_windows	0.2.4	https://github.com/tolkit/fasta_windows
FastK	666652151335353eef2fcd58880bcef5bc2928e1	https://github.com/thegenemyers/FASTK
GenomeScope2.0	2.0.1	https://github.com/tbenavi1/genomescope2.0
Gfastats	1.3.6	https://github.com/vgl-hub/gfastats
GoaT CLI	0.2.5	https://github.com/genomehubs/goat-cli
Hifiasm	0.19.8-r603	https://github.com/chhylp123/hifiasm
HiGlass	44086069ee7d4d3f6f3f0012569789ec138f42b84 aa44357826c0b6753eb28de	https://github.com/higlass/higlass
MerquryFK	d00d98157618f4e8d1a9190026b19b471055b22e	https://github.com/thegenemyers/MERQURY.FK
Minimap2	2.24-r1122	https://github.com/lh3/minimap2
MitoHiFi	3	https://github.com/marcelauliano/MitoHiFi
MultiQC	1.14, 1.17, and 1.18	https://github.com/MultiQC/MultiQC
Nextflow	23.10.0	https://github.com/nextflow-io/nextflow
PretextSnapshot	-	https://github.com/sanger-tol/PretextSnapshot
PretextView	0.2.5	https://github.com/sanger-tol/PretextView
samtools	1.19.2	https://github.com/samtools/samtools
sanger-tol/ascc	0.1.0	https://github.com/sanger-tol/ascc
sanger-tol/blobtoolkit	0.6.0	https://github.com/sanger-tol/blobtoolkit
Seqtk	1.3	https://github.com/lh3/seqtk
Singularity	3.9.0	https://github.com/sylabs/singularity
TreeVal	1.2.0	https://github.com/sanger-tol/treeval
YaHS	1.2a.2	https://github.com/c-zhou/yahs

### Wellcome Sanger Institute – Legal and Governance

The materials that have contributed to this genome note have been supplied by a Darwin Tree of Life Partner. The submission of materials by a Darwin Tree of Life Partner is subject to the **‘Darwin Tree of Life Project Sampling Code of Practice’**, which can be found in full on the Darwin Tree of Life website here. By agreeing with and signing up to the Sampling Code of Practice, the Darwin Tree of Life Partner agrees they will meet the legal and ethical requirements and standards set out within this document in respect of all samples acquired for, and supplied to, the Darwin Tree of Life Project.

Further, the Wellcome Sanger Institute employs a process whereby due diligence is carried out proportionate to the nature of the materials themselves, and the circumstances under which they have been/are to be collected and provided for use. The purpose of this is to address and mitigate any potential legal and/or ethical implications of receipt and use of the materials as part of the research project, and to ensure that in doing so we align with best practice wherever possible. The overarching areas of consideration are:

•     Ethical review of provenance and sourcing of the material

•     Legality of collection, transfer and use (national and international)

Each transfer of samples is further undertaken according to a Research Collaboration Agreement or Material Transfer Agreement entered into by the Darwin Tree of Life Partner, Genome Research Limited (operating as the Wellcome Sanger Institute), and in some circumstances other Darwin Tree of Life collaborators.

## Data Availability

European Nucleotide Archive: Platystomos albinus. Accession number PRJEB75287; https://identifiers.org/ena.embl/PRJEB75287. The genome sequence is released openly for reuse. The *Platystomos albinus* genome sequencing initiative is part of the Darwin Tree of Life Project (PRJEB40665) and the Sanger Institute Tree of Life Programme (PRJEB43745). All raw sequence data and the assembly have been deposited in INSDC databases. The genome will be annotated using available RNA-Seq data and presented through the Ensembl pipeline at the European Bioinformatics Institute. Raw data and assembly accession identifiers are reported in Table 1 and Table 2.

## Author information

Members of the University of Oxford and Wytham Woods Genome Acquisition Lab are listed here: https://doi.org/10.5281/zenodo.12157525.

Members of the Darwin Tree of Life Barcoding collective are listed here: https://doi.org/10.5281/zenodo.12158331.

Members of the Wellcome Sanger Institute Tree of Life Management, Samples and Laboratory team are listed here: https://doi.org/10.5281/zenodo.12162482.

Members of Wellcome Sanger Institute Scientific Operations: Sequencing Operations are listed here: https://doi.org/10.5281/zenodo.12165051.

Members of the Wellcome Sanger Institute Tree of Life Core Informatics team are listed here: https://doi.org/10.5281/zenodo.12160324.

Members of the Tree of Life Core Informatics collective are listed here: https://doi.org/10.5281/zenodo.12205391.

Members of the Darwin Tree of Life Consortium are listed here: https://doi.org/10.5281/zenodo.4783558.
