# Supplementary material for: The genome sequence of a fungus weevil, Platystomos albinus (C.Linnaeus, 1758)
Source: Wellcome Open Res. 2025 Jun 2;10:295. [Version 1] doi: 10.12688/wellcomeopenres.24290.1 (PMC12645098; doi:10.12688/wellcomeopenres.24290.1)
Supplement: Peer review history

# Reviewer report: Reviewer response for version 1

**DOI:** 10.21956/wellcomeopenres.26788.r139017

**Published:** 2025-11-25

**Authors:** Savarimuthu Ignacimuthu, Sivasankaran Kuppusamy

**Affiliations:** 1 St Xavier's College, Palayamkottai, Tamil Nadu, India; 2 Entomology Research Institute, Loyola College Chennai, Tamil Nadu, India

Authors have done the chromosome level genome sequence of fungus weevil, *Platystomosalbinus* (Linnaeus, 1758). They assembled two haplotypes such as chromosome level and scaffold level from the genome sequences. They used appropriate techniques for the High Molecular Weight DNA isolation, library preparation, sequence assembly and curation.

**Minor comments on the manuscript :**

 Authors have assembled two haplotypes one is chromosome level another one is scaffold level. How many SMRT libraries were prepared for the two haplotype assemblies.

 Authors haven’t given sequence annotation details i.e., number of protein coding genes, number of non-coding genes and number of gene transcripts. Any reason? 

 The research article was well prepared and the manuscript meets the necessary scientific standard and is suitable for indexing"

Are sufficient details of methods and materials provided to allow replication by others?

Yes

Is the rationale for creating the dataset(s) clearly described?

Yes

Are the datasets clearly presented in a useable and accessible format?

Yes

Are the protocols appropriate and is the work technically sound?

Yes

Reviewer Expertise:

Molecular biology, phytochemistry

We confirm that we have read this submission and believe that we have an appropriate level of expertise to confirm that it is of an acceptable scientific standard.

# Reviewer report: Reviewer response for version 1

**DOI:** 10.21956/wellcomeopenres.26788.r139024

**Published:** 2025-11-24

**Authors:** Zachary Cohen

**Affiliations:** 1 University of Wisconsin Madison, Madison, Wisconsin, USA

This genome report, for the incredibly diverse *Curculionidae, *offers an important resource for weevil biologists, taxonomists, and geneticists. The report robustly describes the ecological and evolutionary context for this species, as well as a detailed and reproducible methodology. The state-of-the-art approach relies on robust technologies that include HiFi Pacbio long-read sequencing as well as chromatin confirmation via Hi-C. The curatorial and verification steps are the also the industry standard. Another high quality assembly report from DToLC, keep 'em coming!

Are sufficient details of methods and materials provided to allow replication by others?

Yes

Is the rationale for creating the dataset(s) clearly described?

Yes

Are the datasets clearly presented in a useable and accessible format?

Yes

Are the protocols appropriate and is the work technically sound?

Yes

Reviewer Expertise:

Insect evolution, genomics, genetics

I confirm that I have read this submission and believe that I have an appropriate level of expertise to confirm that it is of an acceptable scientific standard.

# Reviewer report: Reviewer response for version 1

**DOI:** 10.21956/wellcomeopenres.26788.r126871

**Published:** 2025-07-28

**Authors:** Maria Antonia Madrid Restrepo

**Affiliations:** 1 KU Leuven, Leuven, Belgium

In this manuscript, Liam M. Crowley and Xavier Richard Badham report the genome assembly of the fungus weevil, P. albinus. They generate a comprehensive reference genome assembly for this species, opening the doors for further research on this beetle. The authors report the presence of 10 chromosomes, plus the X sex chromosome and the mitochondrial genome. 

 I enjoyed the contents of this paper, the authors report in a very detailed manner all the steps for a genome assembly. All steps seem to be highly reproducible, and I have only one editorial comment:

 "The species **if** sexually dimorphic", should be "the species is..."

 Moreover, I would argue that the manual curation section of the manuscript could be more detailed, since the smaller details of the process are not explained, and although manual curation is highly dependent on the genome and the researchers, a better explanation of the steps would be highly appreciated. 

 Besides these two very minor revisions, I am confident that addressing them will further strengthen this manuscript.

Are sufficient details of methods and materials provided to allow replication by others?

Yes

Is the rationale for creating the dataset(s) clearly described?

Yes

Are the datasets clearly presented in a useable and accessible format?

Yes

Are the protocols appropriate and is the work technically sound?

Yes

Reviewer Expertise:

Evolutionary genomics, bioinformatics, population genetics, ecology, biology

I confirm that I have read this submission and believe that I have an appropriate level of expertise to confirm that it is of an acceptable scientific standard.
